# Predictive ability of scores for bleeding risk in heart disease outpatients on warfarin in Brazil

**DOI:** 10.1371/journal.pone.0205970

**Published:** 2018-10-19

**Authors:** João Antonio de Queiroz Oliveira, Antonio Luiz Pinho Ribeiro, Daniel Dias Ribeiro, Vandack Nobre, Manoel Otávio da Costa Rocha, Maria Auxiliadora Parreiras Martins

**Affiliations:** 1 Faculdade de Medicina da Universidade Federal de Minas Gerais, Belo Horizonte, Minas Gerais, Brazil; 2 Hospital das Clínicas da Universidade Federal de Minas Gerais, Belo Horizonte, Minas Gerais, Brazil; 3 Faculdade de Farmácia da Universidade Federal de Minas Gerais, Belo Horizonte, Minas Gerais, Brazil; Maastricht University Medical Center, NETHERLANDS

## Abstract

**Introduction:**

Bleeding is a common complication in patients taking warfarin. We sought to compare the performance of nine prediction models for bleeding risk in warfarin-treated Brazilian outpatients.

**Methods:**

The dataset was derived from a clinical trial conducted to evaluate the efficacy of an anticoagulation clinic at a public hospital in Brazil. Overall, 280 heart disease outpatients taking warfarin were enrolled. The prediction models OBRI, Kuijer et al., Kearon et al., HEMORR_2_HAGES, Shireman et al., RIETE, HAS-BLED, ATRIA and ORBIT were compared to evaluate the overall model performance by Nagelkerke’s R^2^ estimation, discriminative ability based on the concordance (*c*) statistic and calibration based on the Hosmer-Lemeshow goodness-of-fit statistic. The primary outcomes were the first episodes of major bleeding, clinically relevant non-major bleeding and non-major bleeding events within 12 months of follow-up.

**Results:**

Major bleeding occurred in 14 participants (5.0%), clinically relevant non-major bleeding in 29 (10.4%), non-major bleeding in 154 (55.0%) and no bleeding at all in 115 (41.1%). Most participants with major bleeding had their risk misclassified. All the models showed low overall performance (R^2^ 0.6–9.3%) and poor discriminative ability for predicting major bleeding (*c* <0.7), except Shireman et al. and ORBIT models (*c* 0.725 and 0.719, respectively). Results were not better for predicting other bleedings. All models showed good calibration for major bleeding.

**Conclusions:**

Only two models (Shireman et al. and ORBIT) showed at least acceptable performance in the prediction of major bleeding in warfarin-treated Brazilian patients. Accurate models warrant further investigation to be used in similar populations.

## Introduction

Anticoagulation therapy has been proved to be effective in reducing the risk of thromboembolic events. Although vitamin K antagonists, such as warfarin, are widely used for the prevention of these events, bleeding complications are not uncommon [1]. Annual incidence of warfarin-related bleeding varies considerably according to study design and indication, intensity and duration of anticoagulation therapy, ranging from 1.1 to 3.4% for major bleeding [2].

Several methods to estimate the risk for warfarin-related bleeding have been developed, adapted and validated [3–14]. Accurate models for prediction of bleeding risk are useful for medical practices and patient care during oral anticoagulation treatment reducing the risk of life-threatening bleeding complications and potentially improving the efficacy of drug therapy by providing individualized care to patients with higher bleeding risk. However, there is a lack of information about the performance of these models in populations from low and middle-income countries, like Brazil. The aim of this study was to compare the performance of the main models for predicting bleeding risk found in literature in a Brazilian group of outpatients with heart diseases on warfarin.

## Methods

### Study design, setting and participants

A prospective study was performed to compare the predictive ability of nine scores used to evaluate the bleeding risk in patients using warfarin as anticoagulation therapy. The data analyzed in the present study was derived from a clinical trial conducted to evaluate the efficacy of an anticoagulation clinic at a university public hospital located in Southeast Brazil. The main condition for the admission of patients at this clinic is the baseline diagnosis of cardiopathy, such as atrial fibrillation, rheumatic valve disease and Chagas cardiomyopathy. This trial included patients aged 18 years or older presenting a diagnosis of cardiac disease and with at least one indication of long-term oral anticoagulation, such as atrial fibrillation/flutter, mechanical heart valves, history of ischemic stroke/transient ischemic attack or thrombosis. During a one-year follow up, information regarding bleeding episodes was collected from 280 patients included in the trial. Then, we analyzed the performance of nine predictive models for warfarin-related bleeding risk in this population. The trial was approved by the Research Ethics Committee of the Federal University of Minas Gerais (ETIC 376/09) and it was registered at ClinicalTrials.gov (NCT01006486). All study participants signed an informed consent form.

### Variables and classification of bleeding risk

Demographic, clinical and laboratory data were used to calculate the bleeding risk according to the OBRI [5], Kuijer et al. [6], Kearon et al. [15], HEMORR_2_HAGES [7], Shireman et al. [8], RIETE [9], HAS-BLED [10], ATRIA [12] and ORBIT [16] prediction models (Table 1).

**Table 1:** Risk factors and categories proposed by the prediction models for bleeding.

Prediction model	Risk factor	Points for presence	Risk categories
OBRI [5]	Age ≥65 years	1	Low: 0
	Stroke	1	Intermediate: 1–2
	GI bleeding	1	High: 3–4
	Presence of one or more specific comorbid condition: recent MI, Hct <30%, Cr >1.5 mg/dL, diabetes mellitus	1	
Kuijer et al. [6]	Age ≥60 years	1.6	Low: 0
	Female sex	1.3	Intermediate: 1–3
	Malignancy	2.2	High: > 3
Kearon et al. [15]	Age ≥65 years	1	Low: 0
	Stroke	1	Intermediate: 1
	Peptic ulcer disease	1	High: ≥ 2
	GI bleeding	1	
	Abnormal renal function†	1	
	Anemia#	1	
	Thrombocytopenia (platelet count <150,000/μL)	1	
	Hepatic disease*	1	
	Diabetes mellitus	1	
	Concurrent use of antiplatelet drugs	1	
HEMORR2HAGES [7]	Hepatic* or renal disease†	1	Low: 0–1
	Ethanol abuse‡	1	Intermediate: 2–3
	Malignancy	1	High: ≥ 4
	Older age (Age ≥75 years)	1	
	Reduced platelet count or function§	1	
	Rebleeding risk (history of bleeding)	2	
	Hypertension (uncontrolled)||	1	
	Anemia#	1	
	Genetic factors (CYP2C9 polymorphisms)	1	
	Excessive fall risk or neuropsychiatric disease**	1	
	Stroke or TIA	1	
Shireman et al. [8]	Age ≥70 years	0.49	Low: ≤ 1.07
	Female sex	0.32	Intermediate: 1.07–2.19
	Remote bleeding event	0.58	High: ≥ 2.19
	Recent bleeding event	0.62	
	Anemia (Hct <30%)	0.86	
	Diabetes mellitus	0.27	
	Ethanol‡ or drug abuse	0.71	
	Concurrent use of antiplatelet drug	0.32	
RIETE [9]	Recent major bleeding	2	Low: 0
	Cr >1.2 mg/dL	1.5	Intermediate: 1–4
	Anemia#	1.5	High: >4
	Malignancy	1	
	Clinically overt pulmonary embolism	1	
	Age >75 years	1	
HAS-BLED [10]	Hypertension (uncontrolled)||	1	Low: 0
	Abnormal renal function†	1	Intermediate: 1–2
	Abnormal liver function*	1	High: ≥3
	Stroke (particularly lacunar)	1	
	Bleeding history or anemia#	1	
	Labile INRs††	1	
	Age >65 years	1	
	Concurrent use of antiplatelet and/or NSAID drugs	1	
	Ethanol abuse‡	1	
ATRIA [12]	Anemia#	3	Low: 0–3
	Severe renal disease (GFR <30 mL/min)	3	Intermediate: 4
	Age ≥75	2	High: ≥5
	Any prior bleeding	1	
	Diagnosed hypertension	1	
ORBIT [16]	Age ≥75	1	Low: 0–2
	Abnormal haemoglobin/hematocrit‡‡	2	Intermediate: 3
	Bleeding history	2	High: ≥4
	Insufficient kidney function (GFR <60 mL/min/1.73 m2)	1	
	Treatment with antiplatelets	1	

GI, gastrointestinal; MI, myocardial infarction; Hct, hematocrit; Cr, serum creatinine concentration; TIA, transient ischemic attack; INR, international normalized ratio; NSAIDs, non-steroidal anti-inflammatory; GFR, glomerular filtration rate.

* Hepatic disease/abnormal liver function were defined as a serum albumin level <3.3 g/dL or diagnostic of hepatic failure.

^†^ Renal disease/abnormal renal function were defined as a serum creatinine level >2.3 mg/dL or diagnostic of kidney failure.

^‡^ Ethanol abuse was defined as a consumption of >20 units of alcohol weekly.

^§^ Reduced platelet count or function was defined as the presence of thrombocytopenia (platelet count <150,000 μL-1) or the concomitant use of antiplatelet agents.

^||^ Uncontrolled hypertension was defined as a systolic blood pressure >160 mmHg.

^#^ Presence of anemia was defined as a hemoglobin level <12 g/dL in female patients and <13 g/dL in male patients.

** Excessive fall risk was defined as age >60 years, presence of neuropsychiatric disease, impaired mobility or any other factor that predispose to fall.

^††^ Labile INRs was defined as poor time in therapeutic range (TTR <60%).

^‡‡^ Abnormal haemoglobin was defined as a hemoglobin level <12 g/dL in female patients and <13 g/dL in male patients, and abnormal haematocrit was defined as a hematocrit level <36% in female patients and <40% in male patients.

Genetic data required for calculation of bleeding risk by the HEMORR_2_HAGES [7] model was not available. In the risk stratification of the prediction model proposed by the Shireman et al. [8], any previous bleeding events were considered as remote bleeding due to the absence of information about the time of the prior bleeding occurrence. Data concerning previous peptic ulcer disease and drug abuse were not available. For RIETE score [9], recent major bleeding was defined as any prior major bleeding. For all the cases in which data was not available, the risk factors were assigned as absent.

The time in therapeutic range (TTR) was determined by the Rosendaal et al. method [17]. We used the follow-up international normalized ratio (INR) measurements to determine the TTR, considering the INRs from baseline till the first major bleeding event or all the INRs during the follow-up for patients with no major bleeding. In ATRIA [12] and ORBIT [16] we assigned severe renal disease and insufficient kidney function, respectively, as risk factors based on the estimated glomerular filtration rate (eGFR) using the creatinine clearance calculated by the Cockcroft-Gault formula [18].

### Outcomes

The primary outcome variables were the first episodes of major bleeding and clinically relevant non-major bleeding events within one year of follow-up. Although the models have not been originally developed for predicting clinically relevant non-major bleeding and non-major bleeding, this outcome was included as an exploratory endpoint, similar to previous comparative studies found in literature [13,19,20]. The severity of bleeding outcomes was classified according to the criteria recommended from the Subcommittee on Control of Anticoagulation of the Scientific and Standardization Committee of the International Society on Thrombosis and Haemostasis [21]. Major bleeding was defined as a fatal bleeding; and/or bleeding in a critical area or organ (e. g. intracranial, retroperitoneal); and/or bleeding causing fall ≥2 g/dL in hemoglobin level, or requiring transfusion of ≥2 units of red blood cells. Fatal bleeding was defined as a bleeding that directly caused death without any other identifiable cause. Clinically relevant non-major bleeding was defined as any overt sign or symptom of bleeding (e.g., more bleeding than would be expected for a clinical circumstance, including bleeding found by imaging alone) that does not fit the criteria for major bleeding but does meet at least one of the following criteria: requiring medical intervention by a healthcare professional; leading to hospitalization or increased level of care; and/or unscheduled face to face contact with a physician for evaluation. Non-major bleeding was defined as events that do not meet the criteria for major bleeding or clinically relevant non-major bleeding [21].

### Statistical analysis

We used descriptive methods to evaluate baseline characteristics. Continuous variables were tested for normality by Kolmogorov-Smirnov test. Continuous variables are presented as mean ± standard deviation (SD) or median (interquartile range), as appropriate, and categorical variables as percentage. Subgroups were compared by Student t-test or Mann-Whitney U tests, when indicated.

As a measurement of the overall model performance, the Nagelkerke’s R^2^ was estimated to provide the magnitude that the model can predict the outcome of interest. The Nagelkerke’s R^2^ may vary from 0.00 (0.0%) to 1.00 (100%) and a higher number indicates a better predictability of the model. Discriminative ability was measured by the concordance (*c*) statistic, which is equal to the area under the curve (AUC) of a receiver operating characteristic (ROC) for a binary outcome, and varies between no discrimination (0.50), poor (0.51–0.69), acceptable (0.70–0.79), excellent (0.80–0.89), outstanding (0.90–0.99) and perfect discrimination (1.00) [22]. AUC values lower than 0.5 indicates that the model performance is worse than a random assumption. We assessed the calibration of the models by the Hosmer-Lemeshow goodness-of-fit (HL-GOF) statistic. Non-significant HL-GOF statistics indicate a well-fitting model. Observed versus expected probabilities plots were also used to assess calibration for major bleeding outcomes. The models were compared using two different ways: a) quantitative models comparison, using the total risk assessed; and b) qualitative models comparison, using the classification of risk (i.e., low-intermediate and high bleeding risk). Statistical significance was considered when p <0.05. Sensitivity analysis was done assigning as present the factors in which we previously had assigned as absent due to insufficient data to evaluate, i.e. peptic ulcer disease (1 point) in Kearon et al. [15] model, genetic factor (1 point) in HEMORR_2_HAGES [7] and drug abuse (0.71 point) in Shireman et al. [8] model. We also assigned for patients who had history of bleeding, previously assigned as remote bleeding (0.58 point) due to the lack of occurrence time information, as recent bleeding (0.62 point) in Shireman et al. [8] model. All statistical analyses were performed using IBM SPSS Statistics for Windows, Version 21.0 (IBM Corp, Armonk, NY).

## Results

### Study participants’ characteristics and outcomes

The demographic and clinical characteristics of the participants stratified by the severity of the bleeding events are summarized in Table 2. Participants had a mean age of 56.8±13.1 years and they were predominantly female (54.6%). Most common clinical characteristics were atrial fibrillation/flutter (63.3%), hypertension (60.0%), history of non-major bleeding (65.7%) and excessive fall risk (52.9%). Almost a third of participants were diagnosed with Chagas disease. At least one episode of major bleeding occurred in 14 participants (5.0%), clinically relevant non-major bleeding in 29 (10.4%), non-major bleeding in 154 (55.0%) and no bleeding at all in 115 (41.1%). Atrial fibrillation/flutter (85.7%), mechanical heart valve (64.3%), hypertension (64.3%) and previous non-major bleeding (92.9%) were more frequent among the participants who had major bleeding during the follow-up.

**Table 2:** Patients’ characteristics including risk factors proposed by the prediction models with stratification by severity and the absence or presence of the bleeding events.

Characteristics	Total (n = 280)	Major bleeding* (n = 14)	Clinically relevant non-major bleeding* (n = 29)	Non-major bleeding* (n = 154)	No bleeding (n = 115)
Age	56.8 ± 13.1	59.1 ± 15.5	54.0 ± 15.3	55.2 ± 12.5	58.4 ± 14.1
>60 years	111 (39.6)	8 (57.1)	9 (31.0)	53 (34.4)	52 (45.2)
≥65 years	78 (27.9)	9 (64.3)	6 (20.7)	31 (20.1)	44 (38.3)
>65 years	74 (26.4)	9 (64.3)	5 (17.2)	29 (18.8)	42 (36.5)
≥70 years	52 (18.6)	9 (64.3)	3 (10.3)	22 (14.3)	28 (24.3)
≥75 years	23 (8.2)	12 (85.7)	2 (6.9)	7 (4.5)	16 (13.9)
>75 years	21 (7.5)	12 (85.7)	2 (6.9)	7 (4.5)	14 (12.2)
Female sex	153 (54.6)	11 (78.6)	19 (65.5)	98 (63.6)	49 (42.6)
Chagas disease	84 (30.0)	5 (35.7)	6 (20.7)	38 (24.7)	42 (36.5)
Atrial fibrillation/flutter	178 (63.3)	12 (85.7)	17 (58.6)	96 (62.3)	74 (64.3)
Stroke	65 (23.2)	2 (14.3)	3 (10.3)	32 (20.8)	31 (27.0)
Transient ischemic attack	12 (4.3)	1 (7.1)	5 (17.2)	8 (5.2)	3 (2.6)
Mechanical heart valve	88 (31.4)	9 (64.3)	12 (41.4)	62 (40.3)	23 (20.0)
Deep vein thrombosis	16 (5.7)	1 (7.1)	0 (0.0)	8 (5.2)	7 (6.1)
Pulmonary embolism	8 (2.9)	0 (0.0)	2 (6.9)	5 (3.2)	3 (2.6)
Recent myocardial infarction	0 (0.0)	0 (0.0)	0 (0.0)	0 (0.0)	0 (0.0)
Diabetes mellitus	36 (12.9)	2 (14.3)	5 (17.2)	19 (12.3)	14 (12.2)
Hypertension	168 (60.0)	9 (64.3)	17 (58.6)	93 (60.4)	67 (58.3)
Uncontrolled hypertension†	0 (0.0)	0 (0.0)	0 (0.0)	0 (0.0)	0 (0.0)
Malignancy	12 (4.3)	0 (0.0)	0 (0.0)	8 (5.2)	4 (3.5)
History of bleeding	195 (69.6)	13 (92.9)	21 (72.4)	123 (79.9)	64 (55.7)
Previous major bleeding	46 (16.4)	3 (21.4)	4 (13.8)	29 (18.8)	16 (13.9)
Previous non-major bleeding	184 (65.7)	13 (92.9)	19 (65.5)	118 (76.6)	58 (50.4)
Previous gastrointestinal bleeding	37 (13.2)	5 (35.7)	3 (10.3)	26 (16.9)	10 (8.7)
Anemia					
Hematocrit <30%	7 (2.5)	2 (14.3)	1 (3.4)	3 (1.9)	3 (2.6)
Hematocrit <36% in female and <40% in male patients	60 (21.4)	7 (50.0)	9 (31.0)	30 (19.5)	25 (21.7)
Hb <12 g/dL in female and <13 g/dL in male patients	56 (20.0)	6 (42.9)	10 (34.5)	28 (18.2)	23 (20.0)
Hepatic disease or abnormal hepatic function‡	17 (6.1)	2 (14.3)	4 (13.8)	9 (5.8)	7 (6.1)
Renal disease or abnormal renal function					
Cr >1.2 mg/dL	53 (18.9)	2 (14.3)	6 (20.7)	30 (19.5)	21 (18.3)
Cr >1.5 mg/dL	14 (5.0)	0 (0.0)	0 (0.0)	9 (5.8)	5 (4.3)
Cr >2.3 mg/dL	3 (1.1)	0 (0.0)	0 (0.0)	3 (1.9)	0 (0.0)
eGFR <30 mL/min	10 (3.6)	0 (0.0)	1 (3.4)	6 (3.9)	3 (2.6)
eGFR <60 mL/min	71 (25.4)	3 (21.4)	3 (10.3)	31 (20.1)	39 (33.9)
Renal insufficiency	55 (19.6)	4 (28.6)	5 (17.2)	27 (17.5)	25 (21.7)
Reduced platelet count or function§	114 (40.7)	6 (42.9)	14 (48.3)	72 (46.8)	38 (33.0)
Concurrent use of antiplatelet and/or NSAID drugs	92 (32.9)	5 (35.7)	12 (41.4)	62 (40.3)	27 (23.5)
Antiplatelet	88 (31.4)	5 (35.7)	11 (37.9)	58 (37.7)	27 (23.5)
NSAID	5 (1.8)	0 (0.0)	1 (3.4)	5 (3.2)	0 (0.0)
Excessive fall risk||	148 (52.9)	6 (42.9)	13 (44.8)	74 (48.1)	66 (57.4)
Ethanol abuse#	60 (21.4)	3 (21.4)	5 (17.2)	30 (19.5)	28 (24.3)
TTR	62.6 (46.5-76.8)	40.2 ± 22.2	64.5 (46.0–72.5)	62.0 (47.0-73.2)	63.1 (45.8-80.4)
Labile INRs (TTR <60%)	124 (44.3)	11 (78.6)	10 (34.5)	69 (44.8)	52 (45.2)
INR range target					
2.0–3.0	198 (70.7)	9 (64.3)	17 (58.6)	95 (61.7)	95 (82.6)
2.5–3.5	82 (29.3)	5 (35.7)	12 (41.4)	59 (38.3)	20 (17.4)

Values are mean ± SD, median (interquartile range) or n (%). Hb, hemoglobin level; Cr, serum creatinine concentration; eGFR, estimated glomerular filtration rate; NSAIDs, non-steroidal anti-inflammatory; TTR, time in therapeutic range; INR, international normalized ratio.

* Includes patients who had experienced also events of other severity.

^†^ Uncontrolled hypertension was defined as a systolic blood pressure >160 mmHg measured on two occasions.

^‡^ Hepatic disease/abnormal liver function were defined as a serum albumin level <3.3 g/dL or diagnostic of hepatic failure.

^§^ Reduced platelet count or function was defined as the presence of thrombocytopenia or the concomitant use of antiplatelet agents.

^||^ Excessive fall risk was defined as age >60 years, presence of neuropsychiatric disease, impaired mobility or any other factor that predisposes to falls.

^#^ Ethanol abuse was defined as a consumption of >20 units weekly or the presence of alcohol-related health problems.

### Classification of bleeding risk

Most participants were classified in the intermediate risk category using OBRI [5] (56.1%), Kuijer et al. [6] (72.1%) and HAS-BLED [10] (49.6%) while Kearon et al. [15] and HEMORR_2_HAGES [7] prediction models classified the participants mainly in the high risk category (51.4% and 47.5%, respectively). ATRIA [12] model classified the majority of the participants who had bleeding, independently of its severity, and those who did not have bleeding in the low risk category. Among the participants who had major bleeding during the follow-up, the majority were classified in the high risk category by Kearon et al. [15], HEMORR_2_HAGES [7], HAS-BLED [10] and ORBIT [16], as well as those participants who had clinically relevant non-major bleeding, except HAS-BLED [10] and ORBIT [16] models which classified the majority of these participants in the low-intermediate risk categories. None participant who had major bleeding and/or clinically relevant non-major bleeding was classified in the high risk category using Kuijer et al. [6] and RIETE [9] models (Table 3).

**Table 3:** Classification of bleeding risk prediction for the total number of participants and its stratification by severity and by the absence or presence of bleeding events.

Prediction model and risk categories	Total (n = 280)	Major bleeding* (n = 14)	Clinically relevant non-major bleeding* (n = 29)	Non-major bleeding* (n = 154)	No bleeding (n = 115)
OBRI [5]					
Low (0)	107 (38.2)	4 (28.6)	16 (55.2)	67 (43.5)	36 (31.3)
Intermediate (1–2)	157 (56.1)	8 (57.1)	12 (41.4)	78 (50.6)	72 (52.6)
High (3–4)	16 (5.7)	2 (14.3)	1 (3.4)	9 (5.8)	7 (6.1)
Kuijer et al. [6]					
Low (0)	66 (23.6)	2 (14.3)	6 (20.7)	29 (18.8)	35 (30.4)
Intermediate (1–2)	202 (72.1)	12 (85.7)	23 (79.3)	117 (76.0)	76 (66.1)
High (≥3)	12 (4.3)	0 (0.0)	0 (0.0)	8 (5.2)	4 (3.5)
Kearon et al. [15]					
Low (0)	50 (17.9)	1 (7.1)	8 (27.6)	28 (18.2)	19 (16.5)
Intermediate (1)	86 (30.7)	5 (35.7)	8 (27.6)	51 (33.1)	34 (29.6)
High (≥2)	144 (51.4)	8 (57.1)	13 (44.8)	75 (48.7)	62 (53.9)
HEMORR2HAGES [7]					
Low (0–1)	27 (9.6)	1 (7.1)	3 (10.3)	13 (8.4)	13 (11.3)
Intermediate (2–3)	120 (42.9)	5 (35.7)	10 (34.5)	64 (41.6)	53 (46.1)
High (> 4)	133 (47.5)	8 (57.1)	16 (55.2)	77 (50.0)	49 (42.6)
Shireman et al. [8]					
Low (≤1.07)	166 (59.3)	4 (28.6)	16 (55.2)	85 (55.2)	75 (65.2)
Intermediate (1.07–2.19)	110 (39.3)	8 (57.1)	12 (41.4)	66 (42.9)	40 (34.8)
High (≥2.19)	4 (1.4)	2 (14.3)	1 (3.4)	3 (1.9)	0 (0.0)
RIETE [9]					
Low (0)	145 (51.8)	6 (42.9)	12 (41.4)	81 (52.6)	59 (51.3)
Intermediate (1–4)	128 (45.7)	8 (57.1)	17 (58.6)	71 (46.1)	51 (44.3)
(≥4)	7 (2.5)	0 (0.0)	0 (0.0)	2 (1.3)	5 (4.3)
HAS-BLED [10]					
Low (0)	9 (3.2)	0 (0.0)	1 (3.4)	2 (1.3)	6 (5.2)
Intermediate (1–2)	139 (49.6)	4 (28.6)	17 (58.6)	83 (53.9)	50 (43.5)
High (>3)	132 (47.1)	10 (71.4)	11 (37.9)	69 (44.8)	59 (51.3)
ATRIA [12]					
Low (0–3)	211 (75.4)	7 (50.0)	20 (69.0)	122 (79.2)	82 (71.3)
Intermediate (4)	33 (11.8)	2 (14.3)	3 (10.3)	13 (8.4)	20 (17.4)
High (≥5)	36 (12.9)	5 (35.7)	6 (20.7)	19 (12.3)	13 (11.3)
ORBIT [16]					
Low (0–2)	140 (50.0)	3 (21.4)	12 (41.4)	68 (44.2)	68 (59.1)
Intermediate (3)	83 (29.6)	3 (21.4)	11 (37.9)	54 (35.1)	25 (21.7)
High (≥4)	57 (20.4)	8 (57.1)	6 (20.7)	32 (20.8)	22 (19.1)

Values shown are n (%).

* Includes patients who had experienced also events of other severity.

### Comparison of the prediction models

**Fig 1:**
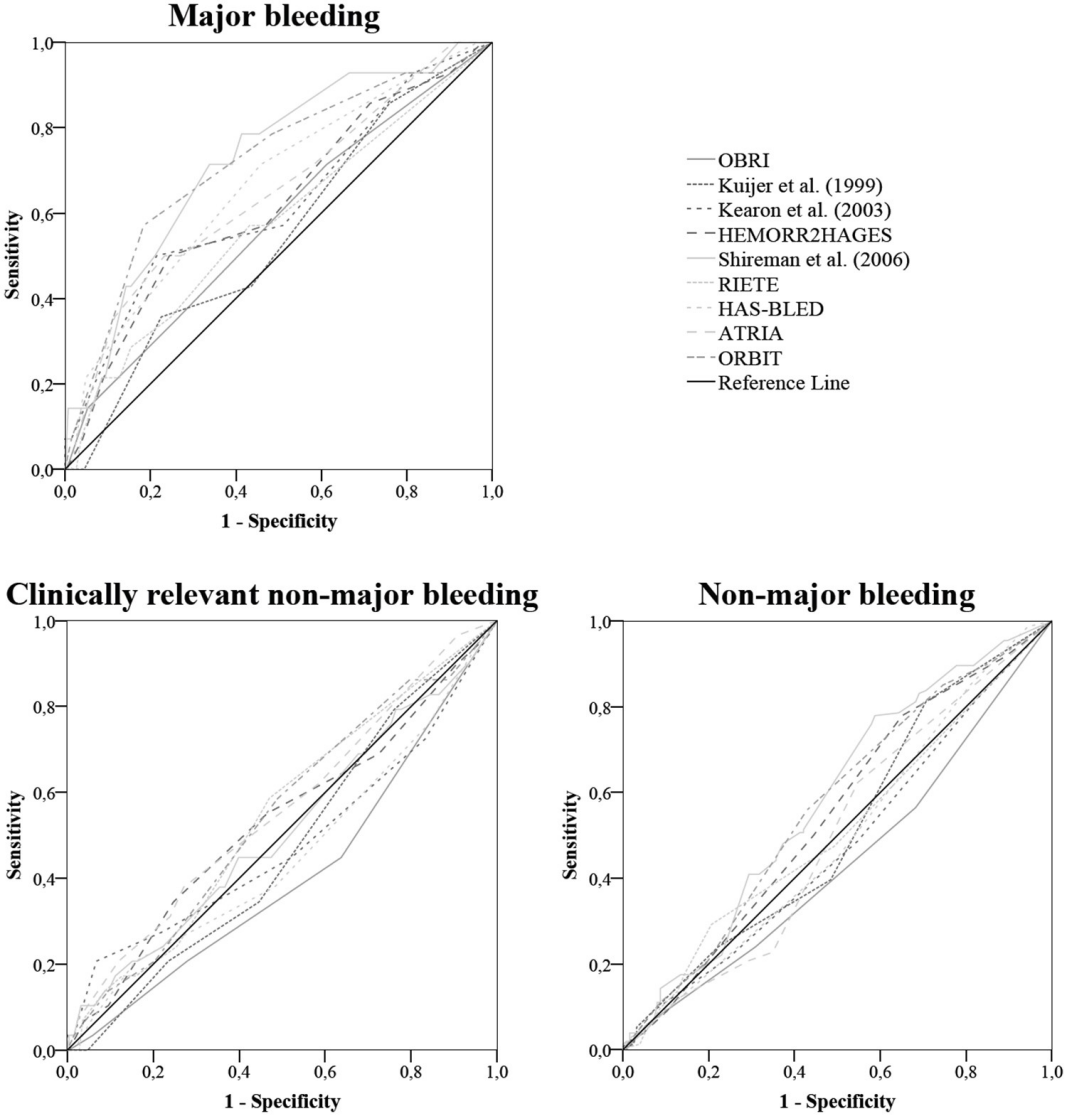
Receiver operating characteristic curves of the models for predicting bleeding risk of major, clinically relevant non-major and non-major bleeding outcomes.

For quantitative models comparison, the discriminative ability of the models for predicting major bleeding, expressed as *c* statistics, ranged from 0.546 to 0.725. Only Shireman et al. and ORBIT models achieved an acceptable level of predictive performance while the others showed poor discriminative ability (*c* index <0.70). All the models showed poor predictive performance when considering clinically relevant non-major bleeding and non-major bleeding as the main outcome, for which the discriminative ability ranged from 0.407 to 0.559, and from 0.438 to 0.582, respectively. There was a significant difference between the discriminative ability of the models for predicting major bleeding and non-major bleeding (p<0.05) (Table 4, Fig 1). The results of discriminative ability in qualitative models comparison were similar to the quantitative approach (S1 Table).

**Table 4:** Discriminative ability of the prediction models (quantitative approach).

Prediction model	Major bleeding		Clinically relevant non-major bleeding		Non-major bleeding	
	AUC	95% CI	AUC	95% CI	AUC	95% CI
OBRI [5]	0.574	0.416–0.733	0.407	0.296–0.518	0.438	0.371–0.505
Kuijer et al. [6]	0.546	0.396–0.697	0.471	0.367–0.575	0.512	0.443–0.581
Kearon et al. [15]	0.624	0.460–0.789	0.479	0.351–0.607	0.476	0.408–0.544
HEMORR_2_HAGES [7]	0.622	0.464–0.780	0.531	0.413–0.650	0.546	0.478–0.615
Shireman et al. [8]	0.725	0.593–0.858	0.510	0.393–0.628	0.582	0.514–0.650
RIETE [9]	0.575	0.411–0.738	0.546	0.438–0.654	0.509	0.441–0.577
HAS-BLED [10]	0.667	0.519–0.815	0.455	0.337–0.574	0.493	0.424–0.562
ATRIA [12]	0.650	0.493–0.807	0.559	0.446–0.673	0.499	0.429–0.568
ORBIT [16]	0.719	0.571–0.866	0.545	0.434–0.655	0.568	0.500–0.636

AUC, area under the ROC curve; CI, confidence interval; SE, standard error. Statistical comparison between the models for predicting major bleeding: p = 0.324; for predicting clinically relevant non major bleeding: p = 0.099; and for predicting non-major bleeding: p<0.05.

All models presented low Nagelkerke’s R^2^ values, which ranged from 0.6% to 9.3% for predicting major bleeding, from 0.0% to 2.0% for clinically relevant non-major bleeding and from 0.0% to 2.7% for predicting non-major bleeding, indicating a poor overall performance. All the models presented no significant difference between their predictive ability and the observed major bleedings (Table 5). Calibration plots showed high concordance between the risk predicted by all models and the observed major bleeding events (S1 Fig). Only Kearon et al. [15] did not fit well for predicting clinically relevant non-major bleeding (p = 0.016) and Kuijer et al. [6] and ATRIA [12] did not fit well for predicting non-major bleeding (p = 0.001 and p = 0.004, respectively) (Table 5). The results of overall performance were similar when the models were compared using the qualitative approach (S2 Table).

**Table 5:** Overall performance and calibration of the prediction models (quantitative approach).

Prediction model	Major bleeding		Clinically relevant non-major bleeding		Non-major bleeding	
	Nagelkerke’s R^2^	HL-GOF χ^2^ (p-value)	Nagelkerke’s R^2^	HL-GOF χ^2^ (p-value)	Nagelkerke’s R^2^	HL-GOF χ^2^ (p-value)
OBRI [5]	0.013	0.510 (0.775)	0.020	1.144 (0.564)	0.014	1.914 (0.384)
Kuijer et al. [6]	0.006	2.159 (0.340)	0.001	3.673 (0.299)	0.005	13.924 (0.001)
Kearon et al. [15]	0.047	4.348 (0.226)	0.000	10.269 (0.016)	0.001	2.158 (0.540)
HEMORR_2_HAGES [7]	0.027	2.957 (0.565)	0.002	2.766 (0.598)	0.007	5.561 (0.234)
Shireman et al. [8]	0.093	3.588 (0.826)	0.001	2.386 (0.935)	0.027	14.095 (0.050)
RIETE [9]	0.012	0.914 (0.822)	0.005	2.355 (0.502)	0.000	5.792 (0.122)
HAS-BLED [10]	0.055	0.384 (0.994)	0.003	3.092 (0.378)	0.000	3.010 (0.390)
ATRIA [12]	0.058	1.809 (0.613)	0.013	1.400 (0.706)	0.002	15.184 (0.004)
ORBIT [16]	0.089	3.398 (0.494)	0.004	4.104 (0.392)	0.016	4.889 (0.299)

HL-GOF, Hosmer-Lemeshow goodness-of-fit.

After sensitivity analysis, no models showed significant enhance of the discriminatory ability or significant improvement in the overall performance. Detailed results of sensitivity analysis can be found in Supporting Information S3, S4 and S5 Tables.

## Discussion

We found that only two (Shireman et al. and ORBIT) of the models tested achieved an acceptable level of discriminative ability for predicting major bleeding while the others showed poor prediction performance. Among the patients who had experienced major bleeding, only the Kearon et al. [15] and HAS-BLED [10] schemes classified the majority in the high-risk category. All the models showed a poor discriminative ability, as reflected by a *c* statistic value lower than 0.7 for predicting clinically relevant non-major and non-major bleeding. Similarly, the overall performance was not good considering the low R^2^ values for all outcomes. All models fit well with the data for predicting major bleeding when the models were compared using quantitative approach.

The majority of the participants who experienced major bleeding were also misclassified by the models in other studies [11,13,14,19]. In the comparative validation study performed by Lip et al. [11], the majority of 136 patients who had major bleeding were stratified in moderate (intermediate) risk category by HAS-BLED, OBRI and Kuijer et al. models and in low risk category by HEMORR_2_HAGES and Shireman et al. models, with none of these patients classified in high risk category by the last one. Similar results were found by Donzé et al. [14] for OBRI, Kuijer et al., Shireman et al. and HAS-BLED. This study also included the RIETE model, which classified the majority of patients into the intermediate category, and ATRIA model, which classified almost the same proportion into the low and the high categories. The high variability in the classification schemes for bleeding risk, especially in participants who had major bleeding, could be explained by differences regarding the clinical characteristics of the participants. For instance, those participants who had mechanical heart valve which requires, in general, a more intense anticoagulation, have an increased risk of bleeding. No models took this fact into account as a risk factor, although the OBRI [5] model included these patients in the validation sample. This could also explain why the majority of the participants who had major bleeding in our study had this condition.

The *c* statistic reported on models’ internal validation studies [5–8,10,12], considering only vitamin K antagonists-treated participants, ranged from 0.632 to 0.82 showing higher results. The discriminative ability achieved an acceptable level only in OBRI [5] (*c* statistic 0.72) and an excellent level in Kuijer et al. [6] (0.82) derivation study. The *c* statistic was not available in the Kearon et al. [15] and the RIETE [9] studies. A more restrict inclusion criteria is one possible reason for the better discriminative ability showed by the models in their original study, as some of them included only patients with venous thromboembolism. This selected group often does not present multiple comorbidities as heart disease patients commonly do. Thus, there would be limitations for its use in populations with other indications of anticoagulation therapy. It is also noteworthy that, in general, all the models had better performance to predict major bleedings when compared to the others levels of severity. This finding was expected since all studied models were developed and validated to predict major bleedings.

Our results reinforce previous results of other comparative studies showing poor overall performance of predictive models [11,13,14,23]. In a recent study with unselected patients receiving oral anticoagulation therapy, Donzé et al. [14] compared seven bleeding risk prediction models and found *c* statistic values ranged from 0.54 to 0.61, concluding that there were no difference between the models and physicians prediction for bleeding risk. In another study, Apostolakis et al. [13] demonstrated only modest performance of HEMORR_2_HAGES, HAS-BLED and ATRIA models in atrial fibrillation patients, with a slightly better performance of the HAS-BLED model for predicting major and any clinical relevant bleeding. However, the *c* statistic ranged from 0.61 to 0.65 for major bleeding and from 0.50 to 0.60 for any clinically relevant bleeding outcome. In a comparative validation, Lip et al. [11] demonstrated a slightly higher *c* statistic value of HAS-BLED (0.66) than four other models (ranged 0.52 to 0.63). Roldán et al. [23], in a two-model comparison study, found a significant better discriminative ability of HAS-BLED over ATRIA when they were analyzed as dichotomized models (0.68 vs 0.59, p = 0.035). Even though some models performed slightly better than others, these studies found *c* statistic values lower than 0.7, indicating poor discriminative ability levels, which were similar to our results for the majority of the models tested, suggesting that these models may not be useful for predicting major, clinically relevant non-major or non-major bleeding. Remarkably, the highest and the only acceptable performance in our study was showed by Shireman et al. and ORBIT models, the latter being the most recent model and one of the simplest to apply in clinical practice.

The discrepant results between the studies of model internal validation and our results could be explained by differences regarding study design and characteristics of the included populations. Some models were developed in studies with short follow-up, such as the studies performed by Kuijer et al. [6], Shireman et al. [8] and RIETE [9] which considered a follow-up period of three months, in contrast with the long-term follow-up performed by OBRI (4 years) [5], Kearon et al. (2.4 years in average) [15], HEMORR_2_HAGES (3 years) [7], HAS-BLED (1 year) [10], ATRIA (6 years) [12] and ORBIT (2 years in average) [16]. The incidence of bleeding events tends to increase as the period of observation becomes longer. This variation could impact on the detection of risk factors for major bleeding and their weight in the development process of the models. The characteristics of the type of patient care may have affected the determination of bleeding risk. OBRI [5], Kuijer et al. [6], RIETE [9] and ORBIT [16] models derived from data of outpatients while HEMORR_2_HAGES [7], Shireman et al. [8] and ATRIA [12] were developed using data of hospitalized patients. On the other hand, HAS-BLED [10] derived from data of ambulatory and hospitalized patients. Most models used database from previous studies designed for other purposes resulting in limitations of the identification of risk factors for bleeding. Regarding our population, Chagas disease is an important and common cause of cardiomyopathy in Brazil, which increases the risk of thromboembolism events. Although this disease has not been considered as a risk factor for bleeding by the predictive models studied, the considerable frequency of chagasic patients presenting major bleeding deserves to be further investigated.

### Limitations and strengths

Some limitations of the present study should be addressed. Firstly, there is a possibility of bias due to the use of a clinical trial database, which was designed for another purpose. There was no data to determine the presence or absence of some risk factors affecting the categorization of bleeding risk. The lack of genetic information could have influenced on HEMORR_2_HAGES [7] classification of risk, although their derivation study did not use this information either. In addition, after performing sensitivity analyses the findings showed to be consistent with those from the primary analysis and lead to similar conclusions. Secondly, as participants with different indications for oral anticoagulation were included, there was no evaluation of the models performance by each indication separately, and many of them differ from the model originally developed. Thirdly, the performance of the models was assessed for predicting not only major, but also clinically relevant non-major and non-major bleeding events, even though they have been developed for predicting major bleeding events only. However, all the models showed poor performance for all outcomes, which demonstrates that they are not good at predicting bleeding events independently of the severity. Finally, we conducted our study at a single university hospital, and the generalizability of our results to other anticoagulation clinics across Brazil and South America remains unclear.

Despite these limitations, this was the first study comparing prediction models of bleeding risk in a population with a low socioeconomic status of a middle-income country and also including a significant proportion of patients presenting Chagas disease or rheumatic valvulopathy. The health systems in low-income and middle-income countries suffer from scarce financial and human resources when compared to high-income countries. Besides, populations from these countries may experience worse conditions of education, health literacy, income and access to high quality health services than high-income countries. Accurate prediction models for bleeding could contribute to the identification of outpatients at higher risks for warfarin complications and to the individualization of the provision of patient care. Consequently, hospital admissions due to bleeding events could be reduced, as well as the costs related to medical interventions. In Brazil, some of the prediction models have been increasingly used to assess the risk of bleeding in patients starting oral anticoagulation. However, our findings suggest that the use of the models tested in clinical practice may not be as useful as expected and highlights the potential need of developing a prediction model derived from similar populations in South America. We recently demonstrated that a dedicated anticoagulation clinic improves the quality and safety of anticoagulation therapy in Brazilian patients [24]. Accurate models to predict bleeding complication could be used to enhance even more the quality of care.

## Conclusions

The comparison of the performance of nine scores for bleeding risk in heart disease outpatients using warfarin in Brazil demonstrated that only Shireman et al. and ORBIT models showed acceptable ability to predict major bleeding, and none showed good performance to predict clinically relevant non-major and non-major bleeding events. The tested models have significant limitations to assess the bleeding risk in warfarin-treated patients and to guide clinical decision on the management of anticoagulation therapy. More accurate models should be investigated to improve the stratification of the bleeding risk and, consequently, to help provide an individualized care to patients, especially in low and middle-income countries from Brazil and other South American countries.

## Supporting information

S1 Table Discriminative ability of the prediction models (dichotomized models). (PDF)

S2 Table Overall performance and calibration of the prediction models (dichotomized models). (PDF)

S3 Table Classification for predicting bleeding risk of the total participants and its stratification by severity and the absence or presence of the bleeding events (sensitivity analysis). (PDF)

S4 Table Discriminatory ability for the prediction models (sensitivity analysis). (PDF)

S5 Table Overall performance and calibration of the prediction models (sensitivity analysis). (PDF)

S1 Fig Plots of observed proportion of major bleeding events in the data versus expected proportion from each model. (PDF)

S1 Dataset All relevant data available. (XLSX)
