# Supplementary material for: Predictive ability of scores for bleeding risk in heart disease outpatients on warfarin in Brazil
Source: PLoS One. 2018 Oct 19;13(10):e0205970. doi: 10.1371/journal.pone.0205970 (PMC6195286; doi:10.1371/journal.pone.0205970)
Supplement: S1 Table — (PDF) [file pone.0205970.s001.pdf]

SUPPORTING INFORMATION

Table S1. Discriminative ability of the prediction models (dichotomized models).

Prediction model	Major bleeding		Clinically relevant non-major bleeding		Non-major bleeding	
	AUC	95% CI	AUC	95% CI	AUC	95% CI
OBRI[5]	0.576	0.417-0.735	0.403	0.293-0.513	0.445	0.378-0.513
Kuijer et al.[6]	0.530	0.390-0.669	0.497	0.392-0.602	0.558	0.490-0.626
Kearon et al.[15]	0.552	0.410-0.694	0.443	0.328-0.559	0.473	0.405-0.541
HEMORR$_2$HAGES[7]	0.569	0.418-0.720	0.554	0.442-0.665	0.528	0.459-0.596
Shireman et al.[8]	0.687	0.537-0.837	0.527	0.414-0.640	0.547	0.480-0.615
RIETE[9]	0.539	0.389-0.690	0.550	0.442-0.657	0.485	0.417-0.553
HAS-BLED[10]	0.633	0.493-0.772	0.450	0.341-0.559	0.486	0.418-0.555
ATRIA[12]	0.646	0.481-0.810	0.542	0.426-0.658	0.461	0.393-0.529
ORBIT[16]	0.717	0.570-0.863	0.539	0.431-0.647	0.554	0.486-0.622

AUC, area under the ROC curve; CI, confidence interval; SE, standard error.
Statistical comparison between the models for predicting major bleeding: $p < 0.05$; for predicting clinically relevant non-major bleeding: $p < 0.05$; and for predicting non-major bleeding: $p < 0.05$.

References

1. Beyth RJ, Quinn LM, Landefeld CS (1998) Prospective evaluation of an index for predicting the risk of major bleeding in outpatients treated with warfarin. Am J Med 105: 91-99.

2. Kuijer PM, Hutten BA, Prins MH, Buller HR (1999) Prediction of the risk of bleeding during anticoagulant treatment for venous thromboembolism. Arch Intern Med 159: 457-460.

3. Kearon C, Ginsberg JS, Kovacs MJ, Anderson DR, Wells P, et al. (2003) Comparison of low-intensity warfarin therapy with conventional-intensity warfarin therapy for long-term prevention of recurrent venous thromboembolism. N Engl J Med 349: 631-639.

4. Gage BF, Yan Y, Milligan PE, Waterman AD, Culverhouse R, et al. (2006) Clinical classification schemes for predicting hemorrhage: results from the National Registry of Atrial Fibrillation (NRAF). Am Heart J 151: 713-719.

5. Shireman TI, Mahnken JD, Howard PA, Kresowik TF, Hou Q, et al. (2006) Development of a contemporary bleeding risk model for elderly warfarin recipients. Chest 130: 1390-1396.

6. Ruiz-Gimenez N, Suarez C, Gonzalez R, Nieto JA, Todoli JA, et al. (2008) Predictive variables for major bleeding events in patients presenting with documented acute venous thromboembolism. Findings from the RIETE Registry. Thromb Haemost 100: 26-31.

7. Pisters R, Lane DA, Nieuwlaat R, de Vos CB, Crijns HJ, et al. (2010) A novel user-friendly score (HAS-BLED) to assess 1-year risk of major bleeding in patients with atrial fibrillation: the Euro Heart Survey. Chest 138: 1093-1100.

8. Fang MC, Go AS, Chang Y, Borowsky LH, Pomernacki NK, et al. (2011) A new risk scheme to predict warfarin-associated hemorrhage: The ATRIA (Anticoagulation and Risk Factors in Atrial Fibrillation) Study. J Am Coll Cardiol 58: 395-401.

9. O'Brien EC, Simon DN, Thomas LE, Hylek EM, Gersh BJ, et al. (2015) The ORBIT bleeding score: a simple bedside score to assess bleeding risk in atrial fibrillation. Eur Heart J 36: 3258-3264.
