# Supplementary material for: Predictive ability of scores for bleeding risk in heart disease outpatients on warfarin in Brazil
Source: PLoS One. 2018 Oct 19;13(10):e0205970. doi: 10.1371/journal.pone.0205970 (PMC6195286; doi:10.1371/journal.pone.0205970)
Supplement: S3 Table — (PDF) [file pone.0205970.s003.pdf]

SUPPORTING INFORMATION

Table S3. Classification for predicting bleeding risk of the total participants and its stratification by severity and the absence or presence of the bleeding events (sensitivity analysis).

Prediction model and risk categories	Total (n=280)	Major bleeding* (n=14)	Clinically relevant non-major bleeding* (n=29)	Non-major bleeding* (n=154)	No bleeding (n=115)
OBRI[1]					
Low (0)	107 (38.2)	4 (28.6)	16 (55.2)	67 (43.5)	36 (31.3)
Intermediate (1-2)	157 (56.1)	8 (57.1)	12 (41.4)	78 (50.6)	72 (52.6)
High (3-4)	16 (5.7)	2 (14.3)	1 (3.4)	9 (5.8)	7 (6.1)
Kuijer et al.[2]					
Low (0)	66 (23.6)	2 (14.3)	6 (20.7)	29 (18.8)	35 (30.4)
Intermediate (1-2)	202 (72.1)	12 (85.7)	23 (79.3)	117 (76.0)	76 (66.1)
High (≥3)	12 (4.3)	0 (0.0)	0 (0.0)	8 (5.2)	4 (3.5)
Kearon et al.[3]					
Low (0)	0 (0.0)	0 (0.0)	0 (0.0)	0 (0.0)	0 (0.0)
Intermediate (1)	50 (17.9)	1 (7.1)	8 (27.6)	28 (18.2)	19 (16.5)
High (≥2)	230 (82.1)	13 (92.9)	21 (72.4)	126 (81.8)	96 (83.5)
HEMORR$_2$HAGES[4]					
Low (0-1)	6 (2.1)	0 (0.0)	1 (3.4)	3 (1.9)	3 (2.6)
Intermediate (2-3)	73 (26.1)	2 (14.3)	8 (27.6)	32 (20.8)	37 (32.2)
High (> 4)	201 (71.8)	12 (85.7)	20 (69.0)	119 (77.3)	75 (65.2)
Shireman et al.[5]					
Low (≤1.07)	85 (30.4)	1 (7.1)	8 (27.6)	31 (21.1)	51 (44.3)
Intermediate (1.07-2.19)	183 (65.4)	10 (71.4)	19 (65.5)	114 (74.0)	62 (53.9)
High (≥2.19)	12 (4.3)	3 (21.4)	2 (6.9)	9 (5.8)	2 (1.7)
RIETE[6]					
Low (0)	145 (51.8)	6 (42.9)	12 (41.4)	81 (52.6)	59 (51.3)
Intermediate (1-4)	128 (45.7)	8 (57.1)	17 (58.6)	71 (46.1)	51 (44.3)
High (≥4)	7 (2.5)	0 (0.0)	0 (0.0)	2 (1.3)	5 (4.3)
HAS-BLED[7]					
Low (0)	9 (3.2)	0 (0.0)	1 (3.4)	2 (1.3)	6 (5.2)
Intermediate (1-2)	139 (49.6)	4 (28.6)	17 (58.6)	83 (53.9)	50 (43.5)
High (>3)	132 (47.1)	10 (71.4)	11 (37.9)	69 (44.8)	59 (51.3)
ATRIA[8]					
Low (0-3)	211 (75.4)	7 (50.0)	20 (69.0)	122 (79.2)	82 (71.3)
Intermediate (4)	33 (11.8)	2 (14.3)	3 (10.3)	13 (8.4)	20 (17.4)
High (≥5)	36 (12.9)	5 (35.7)	6 (20.7)	19 (12.3)	13 (11.3)
ORBIT[9]					
Low (0-2)	140 (50.0)	3 (21.4)	12 (41.4)	68 (44.2)	68 (59.1)
Intermediate (3)	83 (29.6)	3 (21.4)	11 (37.9)	54 (35.1)	25 (21.7)
High (≥4)	57 (20.4)	8 (57.1)	6 (20.7)	32 (20.8)	22 (19.1)

Values shown are n (%).
Sensitivity analysis: Kearon et al.[3] model, peptic ulcer disease risk factor=1 for all patients; HEMORR$_2$HAGES[4], genetic risk factor=1 for all patients; Shireman et al.[5] model, remote bleeding=0.62 if present, and ethanol or drug abuse=0.71 for all patients.
* Includes patients who had experienced also events of other severity.

References

1. Beyth RJ, Quinn LM, Landefeld CS (1998) Prospective evaluation of an index for predicting the risk of major bleeding in outpatients treated with warfarin. Am J Med 105: 91-99.

2. Kuijer PM, Hutten BA, Prins MH, Buller HR (1999) Prediction of the risk of bleeding during anticoagulant treatment for venous thromboembolism. Arch Intern Med 159: 457-460.

3. Kearon C, Ginsberg JS, Kovacs MJ, Anderson DR, Wells P, et al. (2003) Comparison of low-intensity warfarin therapy with conventional-intensity warfarin therapy for long-term prevention of recurrent venous thromboembolism. N Engl J Med 349: 631-639.

4. Gage BF, Yan Y, Milligan PE, Waterman AD, Culverhouse R, et al. (2006) Clinical classification schemes for predicting hemorrhage: results from the National Registry of Atrial Fibrillation (NRAF). Am Heart J 151: 713-719.

5. Shireman TI, Mahnken JD, Howard PA, Kresowik TF, Hou Q, et al. (2006) Development of a contemporary bleeding risk model for elderly warfarin recipients. Chest 130: 1390-1396.

6. Ruiz-Gimenez N, Suarez C, Gonzalez R, Nieto JA, Todoli JA, et al. (2008) Predictive variables for major bleeding events in patients presenting with documented acute venous thromboembolism. Findings from the RIETE Registry. Thromb Haemost 100: 26-31.

7. Pisters R, Lane DA, Nieuwlaat R, de Vos CB, Crijns HJ, et al. (2010) A novel user-friendly score (HAS-BLED) to assess 1-year risk of major bleeding in patients with atrial fibrillation: the Euro Heart Survey. Chest 138: 1093-1100.

8. Fang MC, Go AS, Chang Y, Borowsky LH, Pomernacki NK, et al. (2011) A new risk scheme to predict warfarin-associated hemorrhage: The ATRIA (Anticoagulation and Risk Factors in Atrial Fibrillation) Study. J Am Coll Cardiol 58: 395-401.

9. O'Brien EC, Simon DN, Thomas LE, Hylek EM, Gersh BJ, et al. (2015) The ORBIT bleeding score: a simple bedside score to assess bleeding risk in atrial fibrillation. Eur Heart J 36: 3258-3264.
