# Supplementary material for: Predictive ability of scores for bleeding risk in heart disease outpatients on warfarin in Brazil
Source: PLoS One. 2018 Oct 19;13(10):e0205970. doi: 10.1371/journal.pone.0205970 (PMC6195286; doi:10.1371/journal.pone.0205970)
Supplement: S1 Fig — (PDF) [file pone.0205970.s006.pdf]

SUPPORTING INFORMATION

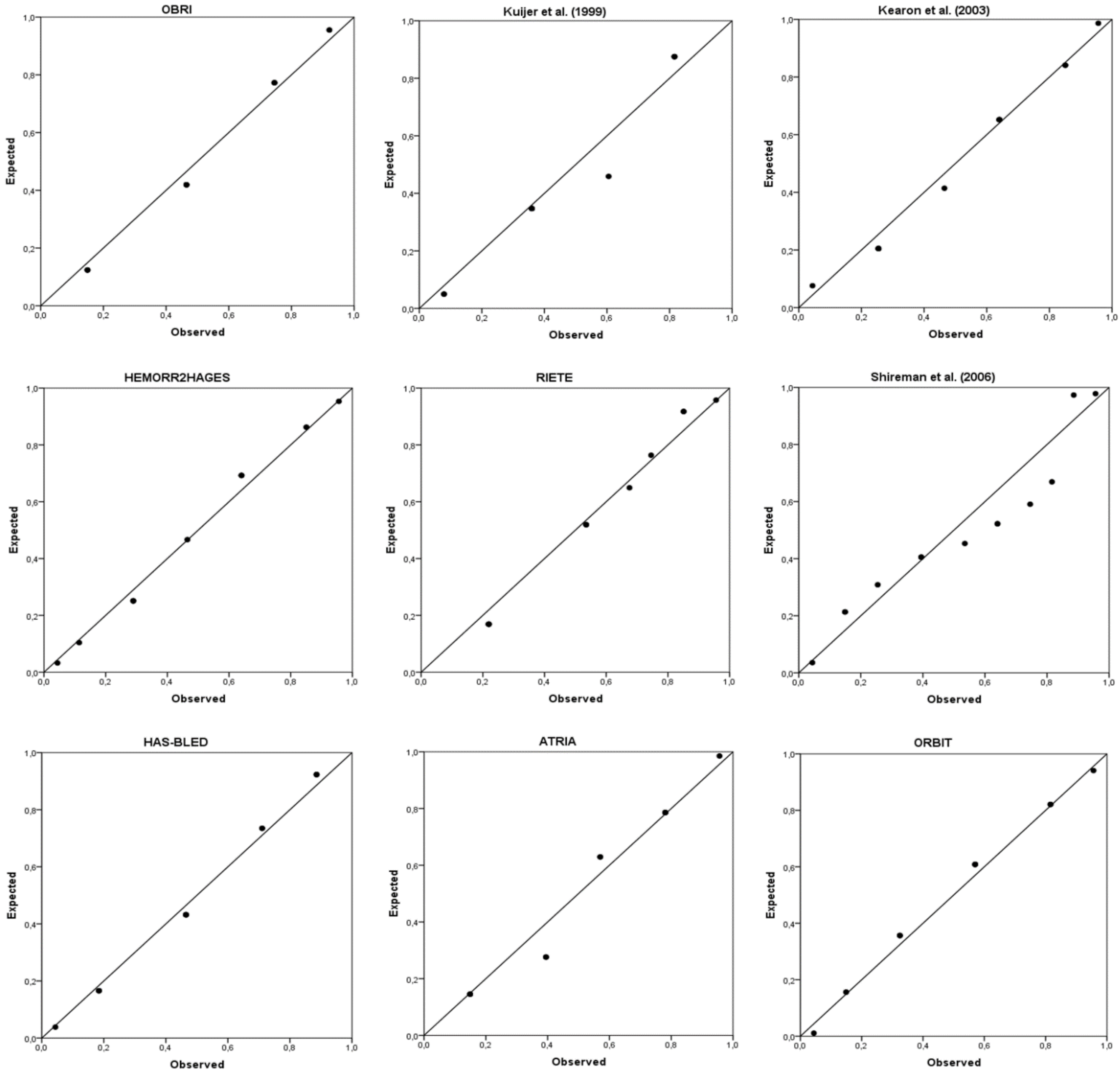

Figure S1. Plots of observed proportion of major bleeding events in the data versus expected proportion from each model.
